# Percutaneous Intervention With Balloon-Expandable Covered Stent for the Treatment of Traumatic Aortic Pseudoaneurysm in a Paediatric Patient

**DOI:** 10.7759/cureus.8453

**Published:** 2020-06-05

**Authors:** Wail Alkashkari, Saad Albugami, Faisal Al-Husayni, Jamilah AlRahimi, Mohammed Althobaiti

**Affiliations:** 1 Cardiology, King Faisal Cardiac Center, King Abdulaziz Medical City, Ministry of National Guard Health Affairs, Jeddah, SAU; 2 Cardiology, King Abdullah International Medical Research Center, King Saud Bin Abdulaziz University for Health Sciences, Jeddah, SAU; 3 Internal Medicine, National Guard Hospital, King Abdulaziz Medical City, Jeddah, SAU; 4 Radiology, King Abdulaziz Medical City, Ministry of National Guard Health Affairs, Jeddah, SAU; 5 Radiology, King Abdullah International Medical Research Center, King Saud Bin Abdulaziz University for Health Sciences, Jeddah, SAU

**Keywords:** injury, aortic trauma, aortic surgery, endograft, aortic stenting, motor vehicle accident, aortic diseases, aortic pseudoaneurysm

## Abstract

In this report, we describe the case of a 13-year-old male who presented to our hospital after sustaining injuries from a motor vehicle accident (MVA). A full-body CT scan revealed multiple injuries, including subgaleal hematoma, hemorrhagic brain contusion, moderate-size aortic pseudoaneurysm with associated mediastinal hematoma, and multiple stable visceral organ lacerations. Additionally, there were numerous fractures, including in the right femur associated with large hematoma. The patient was deemed at high risk for systemic anticoagulation required for an urgent operative aortic repair. Available percutaneous endograft sizes were deemed too large for his descending aortic diameter, and the associated potential risk of vessel injury from the large sheath required to implant the endograft removed this option from consideration. The decision was made to use a balloon-expandable covered stent, which is routinely used to treat coarctation of the aorta (CoA) as a life-saving alternative. A BeGraft aortic stent (Bentley InnoMed, Hechingen, Germany) measuring 16x38 mm was successfully implanted percutaneously with complete exclusion of the pseudoaneurysm without any procedural complications.

## Introduction

Traumatic aortic injury (TAI) is rare in the paediatric population. It is associated with significantly high mortality rates if no intervention is done promptly. Open surgical repair is currently the standard of care, although percutaneous techniques are being investigated [1,2]. In adults, this type of injury is frequently treated percutaneously with self-expanding endograft placement [3,4]. This approach has multiple technical limitations in paediatric patients, specifically the large entry profile of endografts and the inability to adjust the available large endograft diameter with the paediatric small aortic diameter [1-4]. Balloon-expandable covered stents offer a treatment alternative better suited to paediatric patients [5]. The advantages of using such a stent are its small entry profile, easy deliverability, and the fact that it can be expanded to adult size in the future by balloon dilation. It has been used successfully in treating high-risk native/recurrent coarctation of the aorta (CoA) and complications related to CoA intervention such as aortic rupture and pseudoaneurysm formation [5,6]. It is also used successfully in treating late complications associated with the surgical repair of CoA [6]. There are several case reports of the successful use of a balloon-expandable stent in TAI in the paediatric population [7]. In the majority of cases, the Cheatham Platinum (CP) covered stent (B. Braun, Melsungen, Germany) is usually used. Herein, we report the use of the BeGraft aortic stent (Bentley InnoMed, Hechingen, Germany) as an alternative. To our knowledge, our case is the first to report the usage of the BeGraft stent for treating TAI in a paediatric patient.

## Case presentation

**Figure 1:**
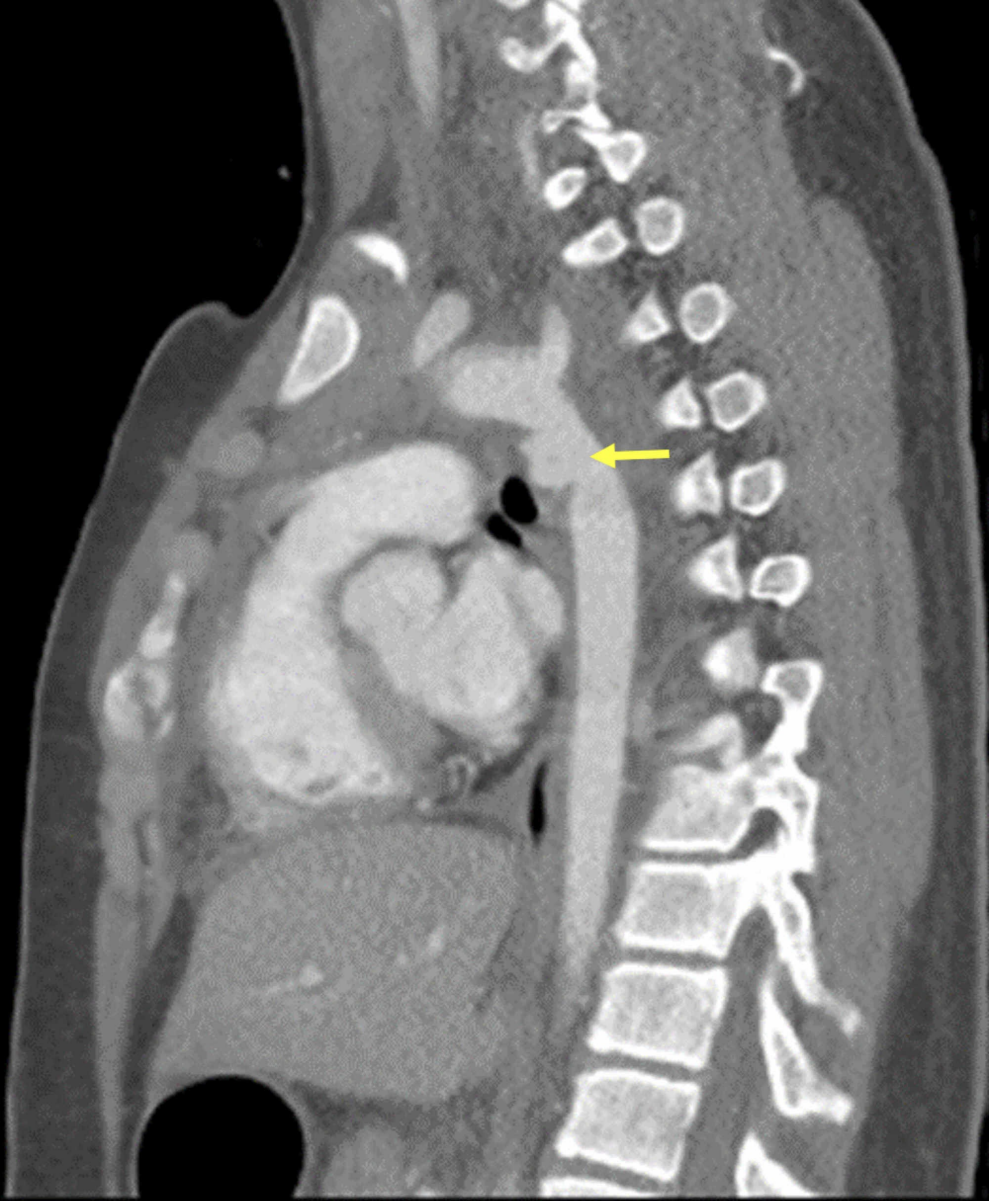
CT of the patient showing the pseudoaneurysm (yellow arrow) CT: computed tomography

A 13-year-old male presented to our hospital after sustaining injuries from a motor vehicle accident (MVA). The patient had bee sitting unrestrained in the front passenger seat of a car when he met with the accident. He had been found unconscious inside the car. Upon arrival in the emergency room, he regained consciousness. As part of the trauma protocol, the patient underwent a full-body CT, which revealed moderate subgaleal hematoma, small hemorrhagic brain contusion, multiple lung contusion, and moderate-size pseudoaneurysm in the descending aorta just distal to the left subclavian artery (LSCA) with associated mediastinal hematoma (Figure 1).

**Figure 2:**
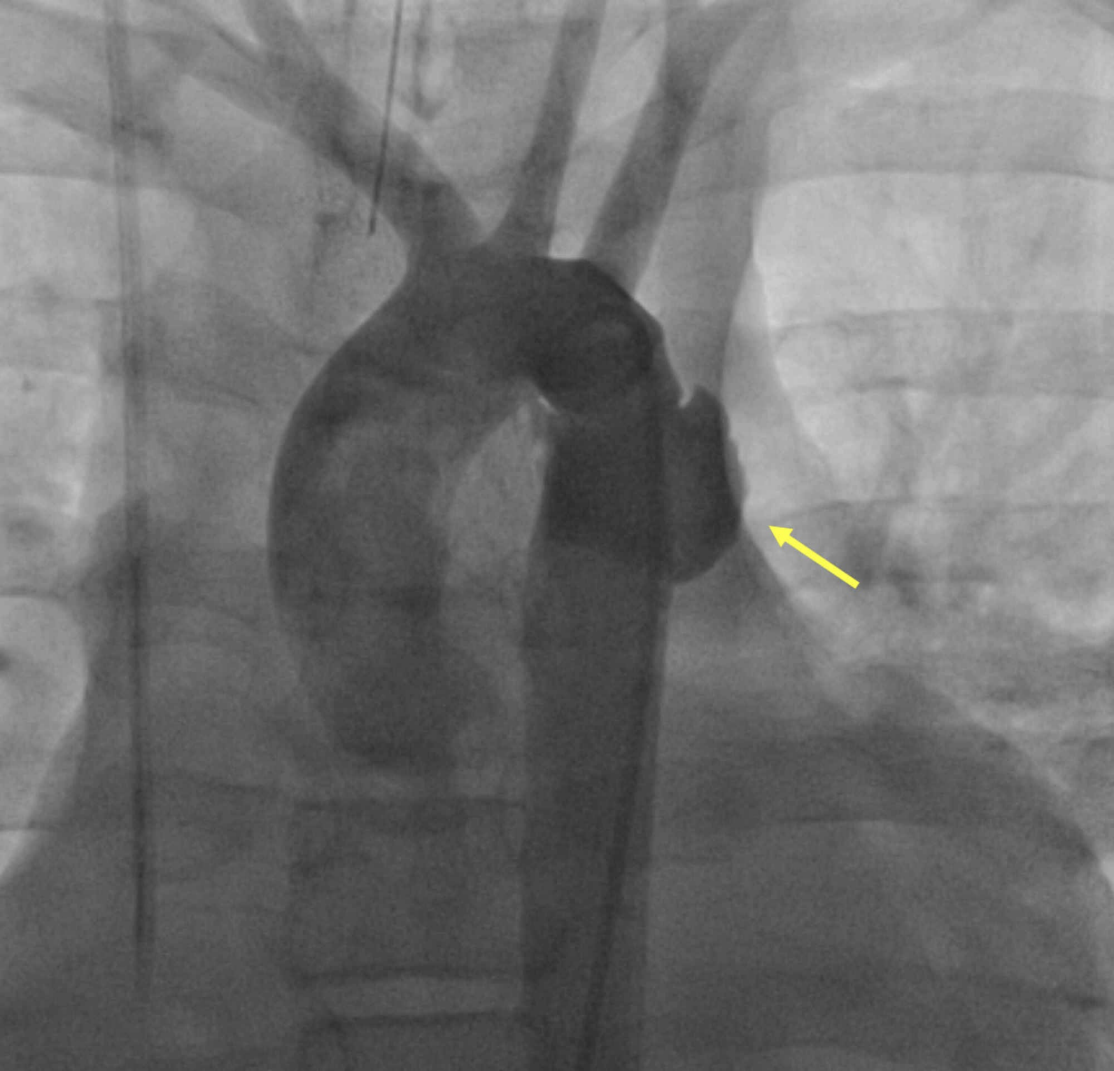
Aortic angiogram showing complex traumatic pseudoaneurysm (yellow arrow)

The CT scan also revealed hepatic, right renal, and splenic lacerations without any evidence of active bleeding. Multiple fractures in the ribs, sternum, right femur, and tibia were also observed. The patient was subsequently admitted to the pediatric intensive care unit (ICU). After a combined surgical and cardiac team discussion, and in the view of other stable lesions, the decision was made to proceed with treating the aortic pseudoaneurysm with impending rupture first. Because of the presence of intracerebral haemorrhage and visceral organ lacerations, the patient was deemed at high risk for systemic anticoagulation required for an operative aortic repair. Available percutaneous endograft sizes were deemed too large for his descending aortic diameter, and the associated potential risk of vessel injury from the large sheath required to implant the endograft removed this option from consideration. The decision was made to attempt percutaneous implantation of a balloon-expandable covered stent across the pseudoaneurysm in the cardiac catheterization laboratory. Under general anesthesia, the right femoral artery access was obtained, and an 11 French (Fr) sheath was inserted. A pigtail catheter was advanced to the transverse aorta, and an angiogram was performed (Figure 2).

**Figure 3:**
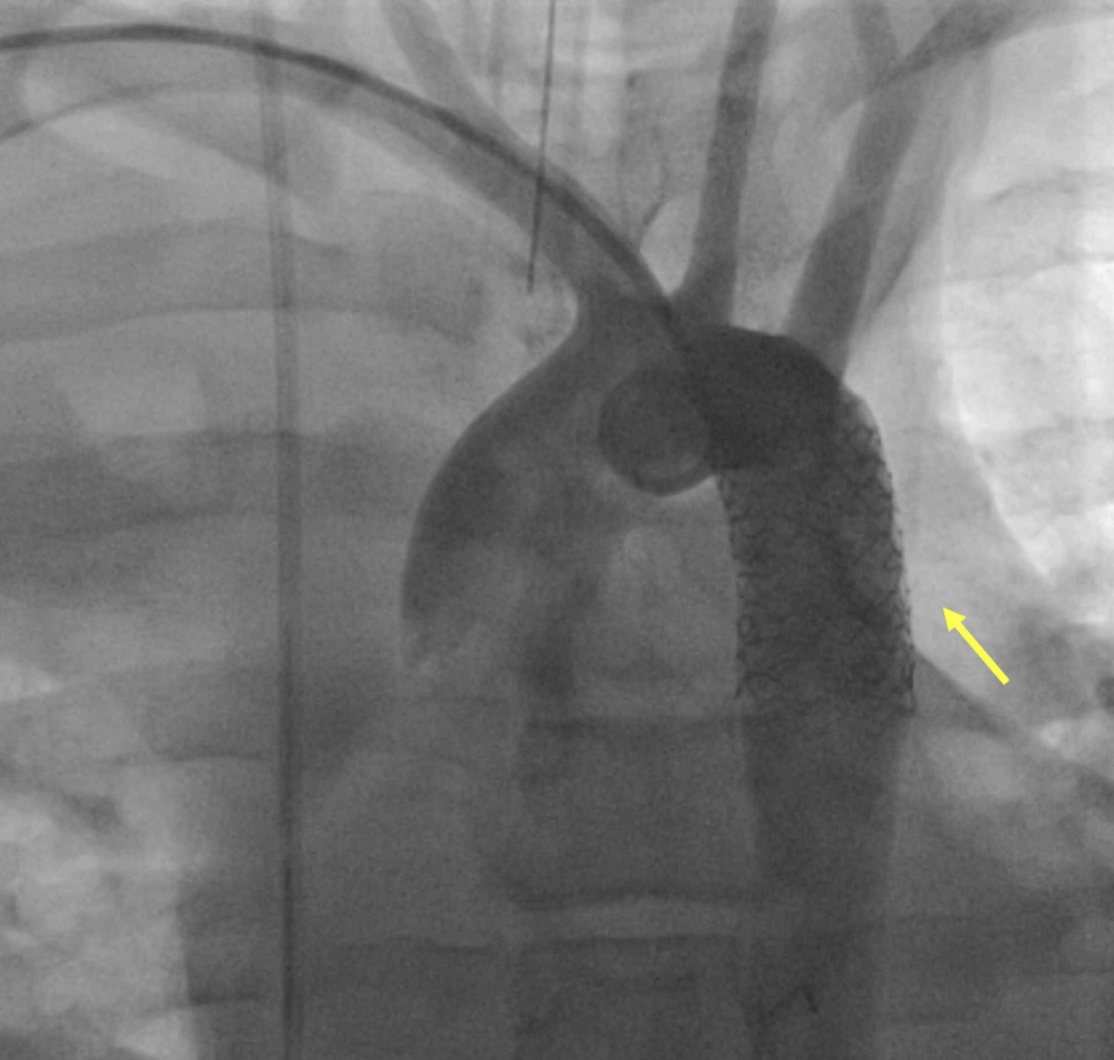
Aortic angiogram after stent placement (yellow arrow)

The angiogram revealed a diameter of 14 mm at the mid-aortic arch, descending aorta immediately after the pseudoaneurysm, and aorta at the level of the diaphragm. The length of the disrupted aortic segment was 22 mm, and it was 10 mm away from the origin of the LSCA. Based on the angiogram measurements, a 16x38 mm BeGraft aortic stent, which is a balloon-expandable covered stent, was used successfully. This was followed by post-stenting dilation with short non-compliant 17 x 30 mm Z-Med balloon (B. Braun, Melsungen, Germany). There was no residual aneurysmal filling, and comparison pressures demonstrated no significant gradient from ascending to descending aorta (Figure 3).

The patient was transferred in stable condition to the pediatric ICU for further management. His post-catheterization course was uneventful. Subsequently, he underwent several orthopaedic procedures until he was discharged home in good condition.

## Discussion

Although paediatric TAI is rare, it presents particularly difficult treatment decisions. Open surgical repair remains the standard of care; however, many patients who have sustained aortic injury have concomitant injuries precluding a safe operative procedure [1,4]. With extensive TAI, surgical options are limited to interposition graft placement, which would require serial replacement with somatic and aortic growth. Endovascular endograft placement is a widely used technique in the adult population and has been used in a smaller number of paediatric patients. The endograft delivery system is large. Patients with small femoral arteries require surgical exposure of the iliac artery before implantation. The endografts are self-expanding, without adjustable diameter; endografts placed in children, therefore, serve as a bridge to a later definitive surgical repair. Balloon-expandable covered stents offer a treatment alternative better suited to paediatric patients [5]. The advantages of using such a stent are its small entry profile, easy deliverability, and the fact that it can be expanded to adult size in the future by balloon dilation without the need for surgery. Disadvantages of such stents include an increased risk of aortic rupture with balloon expansion, stent embolization, and the lack of long-term outcome data.

In comparison to the widely used covered CP stent, the BeGraft aortic stent is characterised by a small flexible profile that requires a delivery system 1-2 Fr smaller than the covered CP stent. Both stents use polytetraﬂuoroethylene (PTFE) as a covering material. The rate of the foreshortening is almost the same for both stents. From our experience with both stents, we believe that the BeGraft stent (cobalt-chromium) is less rigid and opposes better to the wall of the aorta compared to covered CP stent (platinum-iridium). The BeGraft stent is an open-cell design, giving the advantage of re-crossing and open stent cells easily in case if the LSCA is covered inadvertently, while the covered CP stent is a closed-cell design. Both stents can come as pre-mounted stents on the balloon (tri-axial), ready to be introduced to the delivery sheath. The balloon used for the covered CP stent is balloon-in-balloon while the BeGraft stent is mounted on a single balloon [5].

## Conclusions

In this report, we discussed the use of balloon-expandable covered stent in a paediatric patient. Percutaneous implantation of balloon-expandable covered stents may be a safe and effective alternative to open surgical repair or percutaneous endograft implantation for the treatment of TAI in paediatric patients.
